# Consumer Reports of “Keto Flu” Associated With the Ketogenic Diet

**DOI:** 10.3389/fnut.2020.00020

**Published:** 2020-03-13

**Authors:** Emmanuelle C. S. Bostock, Kenneth C. Kirkby, Bruce V. Taylor, Jason A. Hawrelak

**Affiliations:** ^1^Menzies Institute for Medical Research, Hobart, TAS, Australia; ^2^Psychiatry, University of Tasmania, Hobart, TAS, Australia; ^3^Neurology, Menzies Institute for Medical Research, Hobart, TAS, Australia; ^4^College of Health and Medicine, University of Tasmania, Hobart, TAS, Australia; ^5^Australian Research Centre for Complementary and Integrative Medicine, University of Technology Sydney, Ultimo, NSW, Australia

**Keywords:** ketogenic diet, keto flu, symptoms, online forums, consumer reports

## Abstract

**Background:** The ketogenic diet (KD) is a high-fat, low-carbohydrate diet that limits glucose and results in the production of ketones by the liver and their uptake as an alternative energy source by the brain. KD is an evidence-based treatment for intractable epilepsy. KD is also self-administered, with limited evidence of efficacy, for conditions including weight loss, cognitive and memory enhancement, type II diabetes, cancer, neurological and psychiatric disorders. A commonly discussed side effect of KD in media and online forums is “keto flu,” a cluster of transient symptoms generally reported as occurring within the first few weeks of KD. This study aimed to characterize the pattern of symptoms, severity and time course of keto flu as related by users of online forums.

**Method:** Online forums referring to “keto flu,” “keto-induction,” or “keto-adaptation” in the URL were identified in Google. Passages describing personal experiences of keto flu were categorized manually with reference to pattern of symptoms, severity, time course, and remedies proposed.

**Results:** The search criteria identified 75 online forums, 43 met inclusion criteria and contained 448 posts from 300 unique users. Seventy-three made more than one post (mean 3.12, range 2–11). Descriptors of personal experience of keto flu, reported by 101 of 300 users, included 256 symptom descriptions involving 54 discrete symptoms. Commonest symptoms were “flu,” headache, fatigue, nausea, dizziness, “brain fog,” gastrointestinal discomfort, decreased energy, feeling faint and heartbeat alterations. Symptom reports peaked in the first and dwindled after 4 weeks. Resolution of keto flu symptoms was reported by eight users between days 3 and 30 (median 4.5, IQR 3–15). Severity of symptoms, reported by 60 users in 40 forums, was categorized as mild (*N* = 15), moderate (*N* = 23), or severe (*N* = 22). Eighteen remedies were proposed by 121 individual users in 225 posts.

**Conclusions:** Typically, individual posts provided fragmentary descriptions related to the flow of forum conversations. A composite picture emerged across 101 posts describing personally experienced symptoms. User conversations were generally supportive, sharing remedies for keto flu reflecting assumptions of physiological effects of KD.

## Introduction

Ketogenic diets (KD) are low-carbohydrate, high-fat diets which result in elevated levels of ketones in the blood. The metabolic effects of KD mimic starvation (1). Deprivation of glucose is accompanied by hepatic ketogenesis including the production of the ketone bodies acetoacetate, acetone and β-hydroxybutyrate (BHB). Ketone bodies are able to cross the blood-brain barrier, enter neuronal and glial cells and serve as energy substrates for brain cells (2, 3). During ketosis, circulating ketone bodies provide ~70% of the brain's energy requirements (4). High levels of ketone bodies in humans are sustainable for a long period with evidence indicating that it may be tolerated for several years (5).

KD have been used since the 1920s (6) for the treatment of intractable epilepsy and have proven efficacious in controlled trials in children (7–9) and adults (10–12). The mechanisms of action of KD in epilepsy remain unknown, but may include a direct anti-epileptic effect of ketones, in particular acetone (13). The complexities of following KD result in variable compliance to the dietary precepts and the adherence to the diet is not always substantiated by objective measurement of ketones in clinical studies or community samples.

Despite limited high-level evidence for health benefits of KD for conditions other than intractable epilepsy, KD are becoming increasingly popular in the general population. Ketogenic diets, commonly referred to in the popular media as “keto,” have been promulgated for a range of conditions including as a weight loss intervention (14), a cognitive (15) and memory enhancer (16), a treatment for type II diabetes (17), an adjuvant therapy in cancer (18), neurological disorders (19) including migraine (5), and psychiatric disorders (20).

Side effects of KD have received limited systematic attention. In children, a multicenter study of epilepsy inpatients (*N* = 51) reported side effects of KD in 4–8%, including lethargy, dehydration, acidosis, changes in behavior, an increase in infections, constipation, and vomiting. The onset of these side effects occurred in the 2–3 days following initiation of KD and typically resolved within 2 weeks. Side effects were associated with higher levels of ketosis (9). In adults, two reviews (21, 22) and two empirical studies (14, 23) have noted short term adverse effects of KD including gastrointestinal, muscular, and nervous system symptoms.

In popular media, a cluster of transient symptoms/side-effects of KD occurring in the first few weeks of commencing KD are frequently referred to as “keto flu.” Symptoms of keto flu may lead people to cease KD prematurely or be misdiagnosed, leading to unnecessary medical interventions. Information from self-reports on online forums may be useful in characterizing the pattern of symptoms, severity and time course of keto flu and comparing this with clinical research findings. Many individuals following the diet for a variety of indications turn to online forums to share their experiences and receive advice and support from other users. Online forums have been analyzed in relation to various aspects of health including, but not limited to, health and sexuality in adolescent participants (24), breast and prostate cancer (25), and Huntington's disease (26). Campbell and Campbell (27) examined the relationship between ketosis and bipolar disorder in online forum discussions and found a beneficial effect of the diet on mood stabilization. It has been demonstrated that online forums have been used to inform, provide opinions and suggestions (24) and emotional support for participants (25, 26). Online forums are accessible at any time of day and provide the option of anonymity. The aim of this study is to characterize the pattern of symptoms, severity and time course of keto flu as reported in posts by users of online forums.

## Methods

This study was compliant with the Australian National Health and Medical Research Council's National Statement on Ethical Conduct in Human Research (2007). Ethics approval was granted by the University of Tasmania Social Science Ethics Committee (H0018322). This is a descriptive and qualitative assessment of the gray literature and as such does not come under the aegis of either the EQUATOR statement or the PRISMA guidelines.

The study is based on collating and analyzing posts on online forums which address the topic of keto flu. Online forums are comprised of a thread starter established by the user who initiates the forum and posts by users addressing the topic.

Search Strategy: the terms [keto flu, keto-induction or keto-adaptation] and [forum or discussion/s or comments] appear in the URL and [posts] in the text of the forum thread; at least one post in response to a thread starter.

Inclusion criteria: post describes one or more side effects whilst on KD. Exclusion criteria: participants self-reported diabetes; concurrent anabolic steroid use disclosed; reported being on a diet that was not ketogenic; not English language; URL defunct; posts are not in the public domain and may not be viewed without registration or password access; deleted posts as usernames were not visible.

Online forums were systematically identified in the first week of September 2019. Posts, meeting the study criteria, were extracted one forum at a time and anonymized by assigning each forum a sequential numerical identifier, each user an alphabetical identifier, and each post by the same user a numerical identifier (thus 3M7 refers to forum 3, user 14, 7th post). Identifying information, such as username, gender, age, profession, co-morbid medical conditions, and place of origin were not recorded. All names and other information pertaining to individuals were redacted from the text of the posts. Raw data was stored in a password protected database.

Descriptions of keto flu and associated symptoms, severity, time course, and remedies proposed by users were recorded for analysis. A glossary of symptoms was compiled matching investigator defined symptom terms with corresponding verbatim descriptions of symptoms in the posts. For example, verbatim text excerpts in posts, such as “not hungry” and “I didn't feel like eating” would be coded against the investigator defined symptom “decreased appetite.” Since the verbatim text is potentially identifying of individual posts it is not reported in this publication.

The time course of symptoms since starting the diet was noted as occurring on days specified by the user, for example “on days 3–7” was recorded as an occurrence on each of days 3, 4, 5, 6, 7. Where users indicated timing in weeks or months these were recorded against the corresponding number of days, for example 1 week recorded against day 7 and 5 months against day 150.

The severity of symptoms of keto flu as described in the user posts were ranked as mild, moderate or severe. For all symptoms, verbatim descriptions from posts relating to severity were extracted and listed in a Microsoft Excel column, initially in an arbitrary order of severity. Independent raters (EB, KK) then re-ordered entries iteratively, ranking them as less to more severe descending using a sort function. Thesaurus synonyms of mild, moderate or severe were then used to determine cut-off points.

Comments regarding time course and proposed remedies were compiled.

## Results

The search criteria identified 75 online forums, 32 were excluded [diet not ketogenic (10), diabetes (9), not online forum (4), URL defunct (4), not English (3), anabolic steroid use (2)]. Hence, 43 online forums each with a thread starter on the topic of keto flu/induction/adaptation were included in the analysis. These 43 online forums contained a total of 448 posts, attributed to 300 users (unique usernames). Seventy-three users made more than one post (mean 3.12, range 2–11), including six posts by two usernames that appeared on more than one forum.

Descriptors of personal experience of keto flu were recorded by 101 of 300 users and included 256 verbatim symptom descriptions comprising 54 discrete symptoms (see list of the 54 symptoms and counts in Table 1).

**Table 1:** Glossary of symptoms of keto flu derived from online forum posts, with counts and percentages.

**Symptom**	**Count**	**%**
Flu	45	44.6
Headache	25	24.8
Fatigue	18	17.8
Nausea	16	15.8
Dizziness	15	14.9
Brain fog	11	10.9
Gastrointestinal discomfort	11	10.9
Decreased energy	10	9.9
Feeling faint	8	7.9
Heartbeat alterations	6	5.9
Sore throat	6	5.9
Decreased appetite	5	5.0
Shaking	5	5.0
Body aches	4	4.0
Cravings	4	4.0
Cramping	3	3.0
Hunger	3	3.0
Increased thirst	3	3.0
Insomnia	3	3.0
Irritability	3	3.0
Muscular soreness	3	3.0
Muscular weakness	3	3.0
Panic	3	3.0
Sluggish	3	3.0
Anxiety	2	2.0
Bloated	2	2.0
Dehydrated	2	2.0
Diarrhea	2	2.0
Dry mouth	2	2.0
Fever	2	2.0
Joint pain	2	2.0
Lethargic	2	2.0
Listlessness	2	2.0
Weak fingernails	2	2.0
Acne	1	1.0
Asthma	1	1.0
Chills	1	1.0
Constipation	1	1.0
Coughing	1	1.0
Decreased motivation	1	1.0
Depressed mood	1	1.0
Derealization	1	1.0
Ears blocked	1	1.0
Mental confusion	1	1.0
Migraine	1	1.0
Muscle tightness	1	1.0
Phlegm in throat	1	1.0
Photophobia	1	1.0
Restlessness	1	1.0
Run down	1	1.0
Runny nose	1	1.0
Sinus pressure	1	1.0
Tingling in head	1	1.0
Vomiting	1	1.0

Severity of symptoms was reported by 60 users in 40 forums and was categorized as being either mild (*N* = 15), moderate (*N* = 23), or severe (*N* = 22).

Eighteen remedies were proposed in a total of 225 comments by 121 users (see Table 2).

**Table 2:** Remedies for keto flu proposed by users online forum posts, and counts.

**Remedy proposed**	**Count**
Increase sodium intake	58
Supplement with electrolytes	38
Drink broth (including bone broth, stock cubes)	27
Increase magnesium	25
Increase potassium	24
Increase dietary fats (including avocado, MCT, olives, butter, nuts, fat bombs)	14
Increase water intake	14
Drink pickle juice	9
Drink sugar-free sports drinks	9
Increase micronutrients (includes zinc, vitamin B complex)	7
Increase fiber intake (includes dietary fiber from vegetables and fiber supplements)	4
Increase protein intake	3
Include herbal supplements (includes turmeric)	2
Drink apple cider vinegar	1
Include collagen supplementation	1
Increase fruit intake (berries)	1
Increase carbohydrates	1
Decrease carbohydrates	1

**Figure 1:**
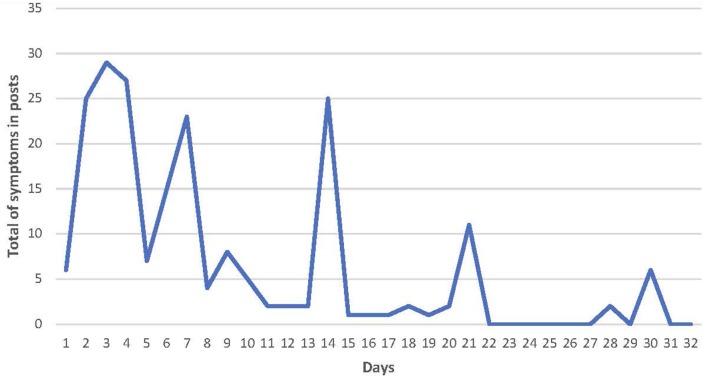
Histogram of the reported time course of onset of symptoms of keto flu.

There were 211 reports of a total of 46 symptoms that indicated the time of occurrence of keto flu in relation to commencement of KD. These reports were in 56 posts by 56 users and ranged from 1 day to 5 months (median 9.5 days, IQR 5.25–20.25) after commencing KD. All but four of the symptom time course reports were between days 1 and 32, the outliers were single complaints of headache (8 weeks), flu (42 days and 4 months) and weak fingernails (5 months). The overall pattern of symptom reports between days 1 and 32 is presented in Figure 1. As noted in the method there are spikes at days 7, 14, 21, and 28 where symptoms were reported at week intervals.

Timing of resolution of keto flu symptoms was reported by eight users as occurring between days 3 and 30 (median 4.5, IQR 3–15). Confirmation of ketosis by urine testing with KETOSTIX was reported by six users, consistent with two being in nutritional ketosis.

## Discussion

The present study examined the pattern of symptoms, severity and time course of keto flu as reported by users of online forums. The forums included were explicitly on the topic of keto flu. The content reflected the conversational nature of such forums where discussion is led by individuals posting specific comments which are typically fragmentary in nature, often relating to comment on a single symptom. However, when examining the range of comments in a given thread and the multiplicity of threads across the 43 forums included, a more composite picture emerges. Personal experience of keto flu was reported by approximately one third of the online forum users. Reported timing of symptoms was typically within several days of starting KD, peaked in the first week and dwindling after 4 weeks. The range of symptoms reported in online forums compares with Harvey's RCT of KD with and without dietary supplementation with medium-chain triglycerides (MCT) in healthy adults (*N* = 28), examining the effect of MCT on the incidence and duration of keto-induction (23). The authors used a 5-point Likert scale to assess the occurrence of symptoms/effects of KD in a period of 20 days. Symptom improvement was observed by day four of commencing the diet. BHB levels were significantly affected by MCT supplementation and participants in that group reached ketosis 2 days in advance of those supplemented by long-chain triglycerides (LCT). Improved symptoms of keto-induction were associated with increased BHB levels achieved through MCT supplementation. The symptoms reported by both Harvey and online forum users were headache, constipation, diarrhea, stomach, or intestinal pain, intestinal bloating, muscle cramps, muscle weakness, and difficulty concentrating. However, in the online forum data only three of these eight symptoms were reported at a frequency of more than 3%, these were headache (24.7%), difficulty concentrating/brain fog (10.9%), stomach or intestinal pain/gastrointestinal discomfort (10.9%). Further, the open-ended nature of the online forum discussions yielded a number of symptoms (Table 1) not included in the closed questions in Harvey's questionnaire. Additional symptoms comprising more than 3% of symptom reports by individual users were flu (44.5%), fatigue (17.82%), nausea (15.8%), dizziness (14.8%), decreased energy (9.9%), feeling faint (7.92%) and heartbeat alterations (5.9%), sore throat (5.9%), decreased appetite (4.9%), shaking (4.9%), body aches (3.9%). In addition to these higher frequency symptoms, the users' online posts reported a further 40 symptoms as being associated with keto flu. Further, two symptoms identified in Harvey's study but not reported by online users were halitosis (bad breath) and skin rash, which were also reported in the KD arm (*n* = 60) of Yancy's RCT in obese adults in a 24-weeks dietary trial (14).

Clinical trials in adults have used the terms “keto-induction” and “keto-adaptation” to describe the time periods of interest in the development and resolution of side effects of KD. Keto-induction refers to immediate effects within several days whereas keto-adaptation is used to refer to multiple organ homeostasis to using ketones as the primary source of energy (28, 29). However, the terms have been used somewhat interchangeably. Keto-adaptation occurs after several weeks of KD and it is hypothesized that it takes months to reach an adequate and stable level of ketones (29). Sherrier and Li (29) also noted that there is currently no quantitative marker for successful keto-adaptation however, in future, this may be demarcated by the time course of changes in circulating ketone bodies, fat and carbohydrate metabolites, muscle glycogen and uric acid. The time course of symptoms reported by the online forum users suggest that side-effects peak in the first 7 days of KD and steadily attenuate over the first month. This is in keeping with their being no arbitrary distinction between the concepts of induction and adaptation. The remedies for keto flu proposed by online forum users are indicative of popularly held beliefs regarding the etiology of keto flu (as summarized in Table 2) and were centered on maintaining hydration and on correcting electrolyte imbalances. It is noted that these remedies and indeed the indications for KD, in so far as these are reported by online forum users are not evidence-based but indicate what is being promulgated by online forum users with some personal experience. Comments were generally pragmatic and there was little discussion of a theoretical or philosophical nature, however this may reflect the focus of the included online forums, which was on the manifestations of keto flu and what to do about it.

Generalizability of the results to the broader population of those adhering to KD is limited by a number of sources of potential bias. There is no comparison group who have commenced KD but are not active in the online forums, this may introduce selection bias based on different characteristics of users and non-users of online forums. Participants were excluded if they self-reported either diabetes or concurrent anabolic steroid use, however no objective measure was available to confirm these exclusions and the majority of forum users did not disclose the reason they were on KD. Whether online forum users had a formal diagnosis and/or were undertaking the diet under medical supervision is also not verified and may have a bearing on results. For example, users may have discussed potential side effects previously with a prescriber of the diet. Symptoms also cannot be attributed to any underlying diagnoses rather than the diet *per se*. It is also not possible to characterize online forum users with respect to variables such a weight, age or race, which may influence the side effect profile. Other factors may introduce measurement bias, as follows. Online user comments are taken at face value. Posts are essentially anonymous and participation is dependent on the motivations of the user, their access to the relevant technology, and in this instance their ability to express themselves in the English language. The experiences of online forum users may not be representative of the larger pool of individuals on KD and they may differ in their likelihood to report negative outcomes. There is also no objective measure of adherence to the dietary precepts of KD or of the establishment of ketosis that are commonly reported in user forums. There is also no objective measure of adherence to the dietary precepts of KD or of the establishment of ketosis that are commonly reported in user forums. Even if such measures were available there is currently no internationally agreed protocols on what constitutes KD with a variety of interpretations as to the importance of such components as macronutrient, phytochemical, and fiber (29).

Notwithstanding these limitations the study gathers information from relatively open-ended discussions by interested individuals sharing their personal experiences and not constrained by the scope of a formal assessment. As such the symptom patterns yielded may indicate candidate items for future questionnaire-based approaches.

## Conclusions

Reports of personal experiences of keto flu by many individuals suggest that the physiological and perhaps psychological changes associated with KD result in the manifestation of an induction and adaptation related syndrome. It is not clear whether this is to be understood in the form of an illness state produced by nutritional and perhaps immune imbalance, or is indicative of an adaptive bodily process triggered by KD. As the repository of online posts increases it will likely be possible to discern an increasingly informative picture of the effects of KD and the features and mechanisms of keto flu.

## Data Availability Statement

The anonymised and redacted datasets generated for this study are available on request to the corresponding author.

## Ethics Statement

The studies involving human participants were reviewed and approved by University of Tasmania Social Science Ethics Committee (H0018322). Written informed consent from the participants' legal guardian/next of kin was not required to participate in this study in accordance with the national legislation and the institutional requirements.

## Author Contributions

EB, KK, BT, and JH contributed to the methodology, interpretation of results, and drafting of the paper. KK and EB categorized the symptom reports in online forum posts and tabulated the results.

### Conflict of Interest

The authors declare that the research was conducted in the absence of any commercial or financial relationships that could be construed as a potential conflict of interest.
